# Biomechanical and Bio-Inspired Perspectives on Root Amputation in Maxillary Molars: An FEA Study

**DOI:** 10.3390/biomimetics10110778

**Published:** 2025-11-15

**Authors:** Öznur Küçük Keleş, Öznur Eraslan

**Affiliations:** 1 Department of Endodontics, Faculty of Dentistry, Ankara Yıldırım Beyazıt University, Ankara 06220, Türkiye; oznurkucukkeles@aybu.edu.tr; 2 Department of Endodontics, Faculty of Dentistry, Selcuk University, Konya 42250, Türkiye

**Keywords:** bio-inspired load transfer, endodontic microsurgery, finite element analysis (FEA), maxillary first molar, root amputation, tooth biomechanics

## Abstract

This study aimed to evaluate the biomechanics of maxillary first molar teeth following palatal, disto-buccal, and mesio-buccal root amputation. An intact maxillary molar underwent root canal treatment using Reciproc R25 files (VDW, Munich, Germany). The canals were obturated with gutta-percha (DiaDent, Seoul, Republic of Korea) and 2Seal sealer (VDW, Munich, Germany), and the access cavity was restored with composite resin. A high-resolution CBCT scan of an intact maxillary first molar was obtained using a Planmeca Promax 3D Max system (Planmeca Oy, Helsinki, Finland) at 75 kVp and 10 mA. The acquired data were processed in 3D Slicer software (v5.8.0, BSD license, Boston, MA, USA) to segment enamel, dentin, and pulp based on pixel density variations using the three-point cloud method. A baseline intact model and three root-resected models (palatal, disto-buccal, mesio-buccal) were reconstructed in SolidWorks 2021, with resected roots simulated as being sealed with MTA. Finite element analysis was conducted in CosmosWorks to evaluate von Mises stress distribution under a 300 N static occlusal load. Maximum von Mises stresses were detected at occlusal force application sites. Among root dentin tissues, stress values ranked highest after palatal root resection, followed by the mesio-buccal, disto-buccal, and non-resected models. Conclusions: Palatal root amputation of maxillary first molars generated the highest von Mises stresses in root dentin, suggesting a higher biomechanical risk than disto-buccal or mesio-buccal resections.

## 1. Introduction

Modern endodontic treatment approaches enable the long-term preservation of teeth, and the success rate of root canal therapy has been reported to range between 86% and 98% [1]. However, due to various factors, treatment may not always be successful, and in such cases, endodontic microsurgery aimed at re-establishing biomechanical balance may be required. Failure may result from a canal reinfection, inadequate disinfection, intra/extra-radicular foreign materials, or extra-radicular resistant infections in the periapical tissues [2]. Cautious clinical assessment should be made between non-surgical retreatment and surgical approaches, especially for endodontic complications in previously restored teeth.

The functional integrity of the tooth depends not only on pulpal health but also on the biological and mechanical stability of the supporting tissues. Therefore, the long-term success of endodontic treatments is closely related to the preservation of the surrounding periodontal tissues. Diseases affecting periodontal tissue may lead to irreversible destruction of the alveolar bone and loss of the periodontal ligaments, resulting in tooth loss if left untreated [3,4]. In multi-rooted teeth, a condition known as furcation involvement may develop [5,6]. Hermann et al. (1983) [7] reported that more than 30% of the attachment surface has already been lost when furcation is exposed. The limited accessibility of the furcation zone limits the effectiveness of non-surgical periodontal treatments in molars and therefore increases the importance of surgical approaches based on biomimetic tissue preservation principles. [8]. Thus, in advanced cases, root amputation is considered an effective surgical technique that allows for the retention of multi-rooted teeth in the mouth instead of extraction.

The biomimetic approach aims to preserve biological integrity and functional balance by mimicking the functioning of natural structures. The natural load transfer system of the tooth and its supporting tissues forms the basis of this concept. Therefore, applying biomimetic principles in surgical procedures such as root amputation contributes to maintaining mechanical strength and biological harmony.

One of these approaches, root amputation, is a treatment method in which one or more roots of a multi-rooted tooth are removed at the furcation level, while the crown and intact roots can be functionally preserved [9,10]. This technique may be indicated only in cases with limited advanced bone loss in a single root and where other conservative approaches are inadequate. Root amputation can be considered a surgical strategy consistent with biomimetic principles. It aims to maintain the tooth’s naturally preservable parts, keep the functional load transfer within physiological limits, and preserve the biological advantages of the natural tooth instead of an artificial implant.

In addition, cases of moderate or severe furcation involvement where the roots diverge in different directions, an unfavorable proximity of roots to adjacent teeth, root fracture, perforation, caries, root resorption affecting a single root or the furcation area, or cases where endodontic treatment of the involved root canal is technically impossible are among the rational indications for root amputation [11]. Similarly, in cases where implant placement is not feasible due to anatomical limitations (e.g., insufficient alveolar bone height, risk of maxillary sinus perforation, or proximity of the inferior alveolar nerve in the mandible), root amputation is an alternative that provides functional preservation of the tooth [12].

After root amputation, sealing the root surface with the proper material is critical to promote healing. Mineral trioxide aggregate (MTA) is a reliable repair material due to its excellent sealing, biocompatibility, and bioactive properties [13]. Mineral trioxide aggregate (MTA) demonstrates several unique qualities that support its clinical effectiveness in endodontics, especially as a root-end filling material in apical surgical procedures. Its use is associated with a favorable healing response of dentoalveolar tissues, including regeneration of the periapical structures and apical cement formation [14]. These properties make MTA a biologically superior option for retrograde filling after root amputation. In this respect, MTA offers a bio-inspired tissue repair model thanks to its structural integrity and biological response.

The tooth and its supporting structures are recognized as natural biomechanical systems optimized through evolution to distribute occlusal loads efficiently. Such structures embody biomimetic design principles that inspire modern dental materials and surgical techniques [15].

In recent years, Finite Element Analysis (FEA) has been widely used to analyze the biomechanical behavior of teeth and surrounding hard tissues [16,17]. FEA offers the opportunity to study the mechanical behavior of complex biological systems with accuracy comparable to that obtained from data, while avoiding the difficulty in obtaining the data by experimental methods [18,19]. This technique provides valuable insights into how apical root resection or other surgical interventions influence stress behavior in dental structures and the alveolar bone. In this context, Kim et al. [20] investigated the effect of apical filling materials and preparation design on stress distribution in the mesial roots of mandibular molars. They emphasized that adequate root wall thickness and apical sealing are critical for achieving favorable stress distribution. However, no study has comparatively examined the stress distribution in dentin and the surrounding bone tissue after amputating different roots in maxillary first molars.

Understanding how root amputation alters this bio-optimized load distribution can guide the development of biomimetic restorative solutions that emulate natural stress pathways. The palatal, mesiobuccal, and distobuccal roots of the maxillary first molar play different roles in load transfer due to their morphological differences. The palatal root, with its larger volume, provides the main support against occlusal forces, while the buccal roots primarily serve a stabilizing function. Therefore, comparing different roots is of great importance for understanding the effects of root morphology and occlusal load transfer on stress distribution.

Therefore, this study aimed to comparatively evaluate the stress distribution, stress concentration zones, and fracture risk in root dentin and the surrounding bone tissue in endodontically treated maxillary first molars retrogradely filled with Mineral Trioxide Aggregate (MTA) following root amputation, using Finite Element Analysis (FEA). The null hypothesis of this study is that amputating different roots in maxillary molars does not affect the maximum stress values and areas of stress concentration in root dentin and the alveolar bone.

## 2. Materials and Methods

This study was reviewed and approved by the Non-Interventional Clinical Research Ethics Committee of Selçuk University Faculty of Dentistry (meeting no: 2025/79, date: 9 September 2025).

Specimen selection and preparation

This study selected one human maxillary first molar extracted for periodontal reasons, with complete root maturation, and free of caries, cracks, or restorations. To exclude structural defects, the tooth was carefully inspected under x4 magnification using a dental loupe (Zumax Medical Co., Ltd., Suzhou, Jiangsu, China). During the clinical examination of the pulp chamber under magnification, canal orifices were carefully evaluated, and the absence of the second mesio-buccal (MB2) canal was confirmed through periapical radiography and cone-beam computed tomography (Planmeca Oy, Helsinki, Finland) analyses. An endodontic access cavity was prepared using fissure and round diamond burs in a high-speed handpiece under copious water cooling to minimize thermal injury to the dental tissues. Following cavity preparation, the working length was established with a size #10 K-file by advancing the instrument until its tip was visible at the apical foramen and subtracting 1 mm. The mesio-buccal and disto-buccal root canals were prepared using Reciproc R25 instruments (VDW, Munich, Germany) operated in the reciprocation program of an X-Smart rotary motor (Dentsply, Maillefer, Ballaigues, Switzerland). The palatal root canal was prepared with Reciproc R40 instruments (VDW, Munich, Germany). Throughout the instrumentation process, each canal was irrigated with 2 mL of freshly prepared 2.5% sodium hypochlorite (NaOCl) solution to ensure disinfection and dissolution of the organic tissues. After completion of canal shaping, the smear layer was removed by sequential irrigation with 5 mL of 17% EDTA, 5 mL of 2.5% NaOCl, and a final rinse of 5 mL of distilled water. The canals were dried using sterile paper points (Dentsply, Maillefer, Ballaigues, Switzerland). Obturation was performed with R25 gutta-percha cones (VDW, Munich, Germany) for the MB and DB root canals and R40 gutta-percha cones (VDW, Munich, Germany) for the palatal canal, together with 2Seal sealer (VDW, Munich, Germany), employing the cold lateral condensation technique. The access cavity was restored by lining the pulp chamber with an SDR bulk fill flowable composite (Dentsply Sirona, Konstanz, Germany) and subsequently sealing the coronal surface with a composite resin restoration (Clearfil AP-X, Kuraray Noritake Dental Inc., Tokyo, Japan) to ensure coronal tightness.

Standardization of the specimens

Following obturation, the specimen represented a standardized endodontically treated maxillary first molar, prepared under consistent protocols to ensure reliability and reproducibility for subsequent experimental and computational analyses.

A reconstructed model of a maxillary molar that had been endodontically treated without receiving root amputation was initially created as the main model (Model 1—Endodontically treated tooth (not resected)). This reference model was then digitally duplicated, and each duplicate was systematically modified to simulate one of the surgical procedures under investigation. The modifications were confined exclusively to the type and extent of surgical intervention, ensuring that all other anatomical and structural parameters remained consistent across the models.

3D Modeling

The specimen was scanned using a Planmeca ProMax 3-D Max cone beam computed tomography (CBCT) scanner (Planmeca Oy, Helsinki, Finland) operating in high-resolution mode at 75 kVp and 10 mA and providing high-resolution volumetric data for subsequent modeling.

The CBCT data were processed using 3D Slicer (v5.8.0, BSD license, CA, USA) image computing software. Enamel, dentin, and pulp tissue were segmented using gray intensity differences and a three-point cloud method. After segmentation, the acquired data were exported in the Standard Triangle Language (STL) format and imported into SolidWorks 2021 (Dassault Systèmes, Waltham, MA, USA).

**Figure 1:**
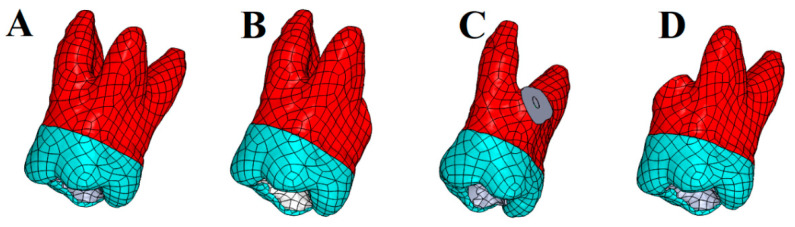
Illustration of different amputation models used in the Finite Element Analysis. (**A**): Intact; (**B**): Palatal root amputation; (**C**): Distal root amputation; (**D**): Mesial root amputation.

After creating a 3D solid model of an endodontically treated maxillary first molar (Model 1) (Figure 1A), three additional models were defined to simulate surgical scenarios:

- Model 2: Resection of the palatal root (Figure 1B);
- Model 3: Resection of the disto-buccal root (Figure 1C);
- Model 4: Resection of the mesio-buccal root (Figure 1D).

In each model, retrograde cavities were prepared on the amputated root surface and simulated by filling them with Mineral Trioxide Aggregate (MTA). In addition, the surrounding supporting tissues were modeled, taking into account the anatomical data reported in the literature: a 0.2 mm thick layer of periodontal ligament surrounding the tooth [21], a 2 mm thick layer of cortical bone placed on top of it, and underlying spongious tissue [22].

**Figure 2:**
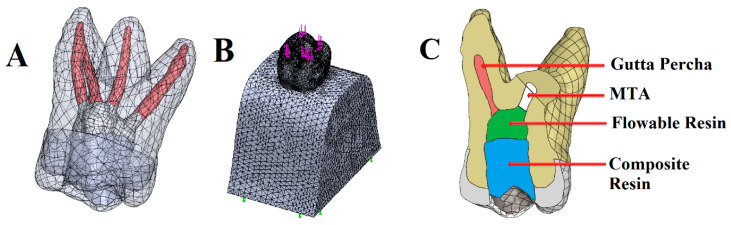
Three-dimensional finite element model of a maxillary first molar. Illustration of tooth components (enamel, dentin, pulp tissue) (**A**). A meshed view of the three-dimensional mathematical model of the maxillary first molar. Purple arrows show load application points at the occlusal surface. Green arrows display the exterior nodes at the bottom surface of the bone structure that were fixed as boundary conditions (**B**). Illustration of different components in the disto-buccal root resection model (**C**).

All materials were assumed to be homogeneous and isotropic. As boundary conditions, nodes at the bottom exterior surface of the bone structure were assumed as fixed in all directions. Convergence analysis was performed. This test was then conducted with a 10% mesh control, growth rate 1.2–1.4, and edge length ≤ 1/5 of the smallest structure circumference, which determined the number of nodes/elements needed to generate models. Then, the finite element models were meshed with tetrahedral quadratic elements (Figure 2). The total number of nodes and tetrahedral solid elements are presented in Table 1. A 300 N vertical occlusal load was applied from contact points at the occlusal surface (Figure 2). The elastic properties of the materials involved in the FEA were acquired from the literature (Table 2).

**Figure 3:**
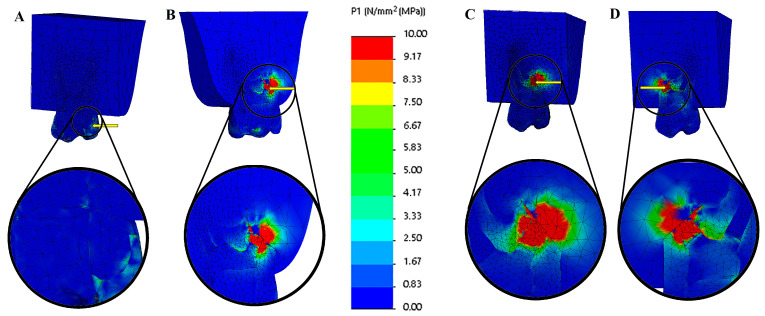
Tensile stress (MPa) distributions of solid models of different options. (**A**) Mesio-distal cross-section view of endodontically treated tooth (not resected), (**B**) bucco-palatal cross-section view of palatal root resection, (**C**) mesio-distal cross-section view of disto-buccal root resection, and (**D**) mesio-distal cross-section view of mesio-buccal root resection. The color scale used in the stress distribution figures numerically represents values in the range of 0–10 MPa. Blue indicates low-stress regions, while red represents high-stress regions.

**Figure 4:**
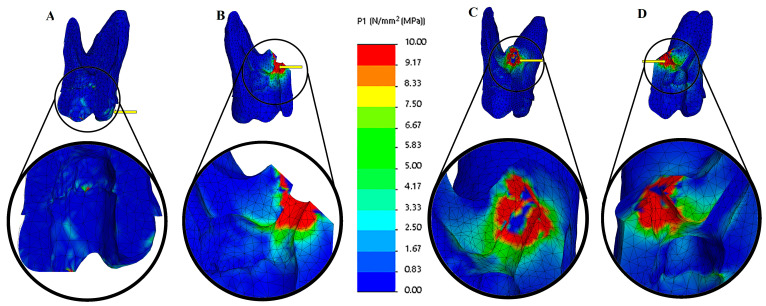
Tensile stress (MPa) distributions of cross-section views of dentin structures. (**A**) Endodontically treated tooth (not resected), (**B**) palatal root resection, (**C**) disto-buccal root resection, and (**D**) mesio-buccal root resection. The color scale used in the stress distribution figures numerically represents values in the range of 0–10 MPa. Blue indicates low-stress regions, while red represents high-stress regions.

Geometric modeling was carried out in SolidWorks 2021 (Dassault Systèmes, Waltham, MA, USA). The models were subsequently imported into CosmosWorks 2021 (Dassault Systèmes) for meshing and finite element analysis. Each component was assigned material properties based on values reported in previous studies to reflect their biomechanical behavior accurately. The tensile stress results are presented as color graphics to enhance the visualization of stress distribution within the models. The scale range was limited to 0–10 MPa for easier comparison of the different models. The mesio-distal and bucco-lingual cross-section view of the 3D main model (Figure 3) and the cross-section view of the root structure (Figure 4) are presented for each restoration option.

## 3. Results

The maximum tensile stress values were observed in the MTA plug material in the resected models. In contrast, in the root-canal-treated tooth model, they were detected in the dentin tissue (Figure 3 and Figure 4). Among the resected models, the palatal root resection exhibited the highest tensile stress values across all tissues (18.33 MPa), followed by the mesio-buccal root resection (14.09 MPa) and the disto-buccal root resection (13.10 MPa). The non-resected tooth model recorded the lowest maximum tensile stress value (8.14 MPa). The highest tensile stress values observed in the enamel, dentin, and root canal filling materials across the different models are summarized in Table 3. In the endodontically treated non-resected tooth model, a nearly homogeneous stress distribution was observed across all dental tissues and the composite restorative material. In contrast, the root canal filling material exhibited the lowest stress concentration (Figure 3).

In all resected models, stress concentrations were observed at the dentin–enamel junction, within the composite restorative material, at the interfaces between dental tissues, and particularly on the root canal filling material adjacent to the resection area. In addition, stress concentrations were also detected in the bone tissue in contact with the resection area (Figure 4).

**Figure 5:**
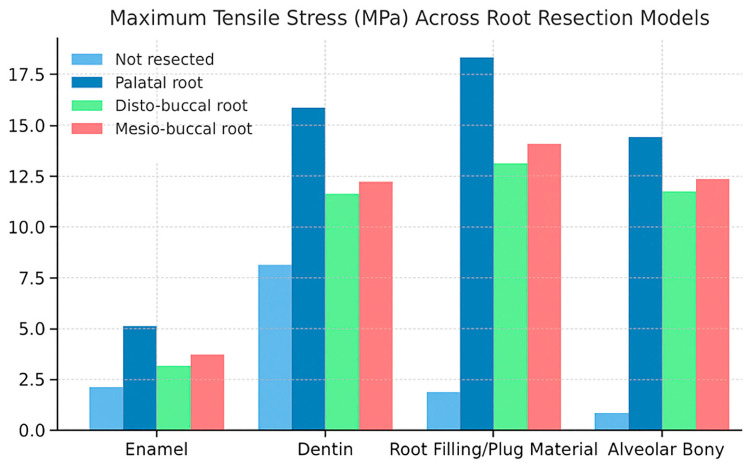
Comparison of maximum stress values in enamel, dentin, filling material, and alveolar bone across different root resection models (non-resected, palatal, disto-buccal, and mesio-buccal).

The highest tensile stress values among all models were observed following palatal root resection. In the non-resected model, the measured stress values in enamel, dentin, root filling material, and alveolar bone were 2.13, 8.14, 1.86, and 0.83 MPa, respectively. After palatal root resection, these values increased to 5.12, 15.86, 18.33, and 14.42 MPa. In the disto-buccal root resection model, the values were 3.13, 11.63, 13.10, and 11.74 MPa, while in the mesio-buccal root resection model, they were 3.72, 12.22, 14.09, and 12.34 MPa, respectively (Figure 5).

The findings indicate that palatal root resection caused a marked increase in stress within both the hard dental tissues and the surrounding supporting structures. The high stress concentration observed, particularly in the root-filling material and alveolar bone, suggests that resection altered the load-transfer balance in these regions, leading to localized stress accumulation. In contrast, the non-resected model exhibited a more homogeneous stress distribution, with a lower risk of structural deformation.

The results indicate that palatal root resection produced the highest tensile stresses in both dentin and surrounding alveolar bone tissue, suggesting a potential biomechanical disadvantage compared to other types of resection.

## 4. Discussion

This study’s primary aim was to investigate the effect of root amputation on root dentin and alveolar bone strength using stress analysis performed using the finite element analysis (FEA) method. The findings indicate that amputation of different roots in maxillary first molar teeth alters the stress distribution pattern in root dentin and surrounding alveolar bone. These results reveal that differences in the location and intensity of stress accumulation vary depending on the amputated root. Therefore, the null hypothesis proposed at the beginning of the study, which stated that “amputation of different roots would not affect stress distribution behavior,” has been rejected.

The present study assessed stress distribution and concentration changes following the amputation of a maxillary first molar’s mesio-buccal, disto-buccal, and palatal roots. The findings demonstrated that resection of all roots significantly altered the stress distribution in the remaining root structures, with stress concentrations particularly observed at the dentin–enamel junction, at the interfaces between the composite filling material and the dental tissues, on the root canal filling material adjacent to the resection area, and within the bone tissue in direct contact with the resection site. These results are consistent with previous reports indicating that alterations in root morphology can lead to marked changes in stress distribution within the tooth structure [30,31]. The findings highlight that the tooth embodies a bio-inspired structural design principle, functioning as a natural load-transfer system; deviations in root morphology disturb this evolutionarily optimized mechanical balance.

The finite element analysis conducted in this study demonstrated that removing any root from an endodontically treated maxillary first molar substantially increased stress accumulation within the remaining tooth structure and adjacent regions. The results revealed that the highest von Mises stress values were detected in the MTA plug material in the resected models. In contrast, the dentin exhibited the greatest stress in the root-canal-treated tooth model (Figure 3 and Figure 4). Among the resection groups, palatal root amputation produced the highest tensile stress across all tissues (18.33 MPa), followed by mesio-buccal root resection (14.09 MPa) and disto-buccal root resection (13.10 MPa). In contrast, the intact, non-resected tooth model demonstrated the lowest and most uniform stress distribution. Following the removal of the largest root, the capacity of the tooth to effectively distribute occlusal forces is substantially diminished, resulting in a reduced cross-sectional area of dentin available to resist these forces [25]. This aligns with fundamental biomechanical principles: if the applied force remains the same but the supporting area is reduced, the pressure (stress) on remaining structures rises (per the relation P = F/A) [32]. The finding that the intact control model had the most homogeneous and lowest stress distribution further supports the notion that any removal of the tooth structure tends to weaken the tooth and elevate internal stresses [25]. Santos-Filho et al. demonstrated that substantial dentin removal compromises root integrity, resulting in increased levels of tensile stress [33]. This study demonstrates that after root resection, maximum stresses concentrate along the amputation line, material continuity is disrupted, and replacement with different tissue (bone) results in the formation of an artificial load transfer pathway. This change indicates a disruption of the tooth’s natural “biomimetic engineering design.”

Our results are consistent with and expand upon previous FEA studies of endodontic surgical scenarios [25,34,35]. Aslan et al. (2024) [25], for example, compared different surgical interventions in a mandibular molar and found that eliminating an entire root (root amputation) led to substantially higher dentinal stress compared to a minor apical resection. In their simulations, root-amputated models showed roughly double the peak stress of a conservatively resected model [25]. Their analysis attributed the stress increases to the loss of supporting root mass and the consequent reduction in load-bearing area, corroborating our explanation [25]. In other words, removing significant dentin weakens the tooth and concentrates stress, precisely what our maxillary molar models demonstrated, especially in the palatal root removal scenario.

One of the aspects of this study that makes it original is the comparative evaluation of different root amputation sites in maxillary molars. Although previous works in the literature have compared root amputation with other surgical procedures, data on which root removal yields the most unfavorable biomechanical outcomes have remained limited [25,34,35,36]. In clinical practice, the palatal root is removed only when mandatory, and the findings obtained from this study support this clinical approach from a biomechanical perspective. The palatal root is the biggest and most resistant of the maxillary molar teeth; amputating this root, resulting in the crown being supported solely by the buccal roots, led to prominent stress increases in our model. In clinical practice, amputation procedures are typically performed on the disto-buccal root and less frequently on the mesio-buccal root, while the palatal root is usually preserved [37]. Our study’s findings indicate that removing the palatal root will result in the highest stress values, and mesio-buccal root resection is associated with higher stress compared to disto-buccal root resection. Therefore, from a biomechanical perspective, when clinically feasible, removing a smaller root appears more favorable for preserving the tooth’s structural integrity compared to losing a larger root. The lowest stress distribution observed in the intact model reveals that while every amputation leads to a certain degree of structural weakening, the severity of this effect varies depending on the type of root removed [33].

Although increased stress values were observed following root resection, root amputation can be considered a long-term treatment option with appropriate case selection and correct restorative approaches. The literature reports that molar teeth undergoing root resection can achieve high survival rates when adequate bone support is preserved. Fugazzotto et al. [38] determined a 15-year success rate of 97% in carefully selected cases and emphasized that this result is comparable to the success rates of implant therapies. Similarly, another long-term 10-year study found an overall success rate of approximately 70%; however, it was noted that survival rates increased significantly when there was more than 50% bone support around the remaining roots [39]. These findings indicate that this study’s high stress values obtained by finite element analysis can be clinically compensated for by adequate periodontal support and appropriate restorative approaches.

This study provides clinically significant insights for decision-making in root resection procedures. The high stress accumulation observed after palatal root resection indicates that this root should be preserved whenever possible. In contrast, distobuccal or mesiobuccal root resections may allow for a more balanced stress distribution. Therefore, restorative planning after resection should include approaches that optimize load distribution, such as occlusal adjustment and splinting.

Although the obtained results are consistent with the literature, the more pronounced increase in stress observed after palatal resection suggests that the maxillary molar possesses a unique biomechanical morphology. This finding highlights the decisive influence of anatomical factors such as root volume and bone support on stress behavior. These biomechanical findings indicate that restorative materials and tooth replacement strategies developed in accordance with biomimetic principles should be designed to replicate the natural load-bearing characteristics of dental roots.

The maxillary first molar was chosen as the study model because of the difficulty in locating and shaping the second MB canal, which, if left untreated, may necessitate surgical intervention of the MB root [40]. Other reasons for selecting the maxillary first molar include the tendency of the MB root to present more apical deltas [41] and the increased complexity of nonsurgical retreatment due to the presence of the second MB canal [40]. The FEA method was employed in this study because it enables the analysis of root amputation as the primary distinguishing factor among the compared teeth. The initial root canal treatment and preparation of all specimens were performed under standardized conditions to minimize variations among the teeth and to ensure that the analysis focused exclusively on the changes related to the extent of root amputation.

Von Mises stress is widely applied in finite element analysis (FEA) as a parameter for evaluating the structural integrity and material strength of dental tissue. It also provides valuable insight into potential sites of mechanical failure [42]. One of the most frequently reported causes of extraction in surgically retreated teeth is vertical root fracture [43,44,45], and von Mises stress distribution can assist in identifying regions of root dentin that may be at risk of failure [46]. For this reason, von Mises stress analysis can provide guidance in determining which areas require structural reinforcement in biomimetic restorative planning.

These biomechanical findings have important clinical implications. Increased stress within the remaining tooth structure may predispose it to crack formation or fracture, particularly under repetitive masticatory forces [47]. Although the 300 N vertical load applied in our study does not fully reflect the dynamic and lateral forces present clinically, supporting root-resected molars with full-coverage restorations, splinting them to adjacent teeth when necessary, and carefully adjusting occlusion may improve their prognosis [37].

The limitations of this research arise from the assumptions inherent to the finite element analysis (FEA) method. Within the scope of this approach, the structures forming the tooth, along with the surrounding dentin and bone tissues, were assumed to exhibit isotropic properties [48,49]; however, since dentin and cortical bone are anisotropic in nature, this assumption may only partially reflect the actual stress distribution. Although adjacent tooth contacts, the periodontal ligament, and bone tissue were included in the model to simulate boundary conditions as realistically as possible, it should be acknowledged that the developed model cannot fully replicate in vivo conditions. Furthermore, the model was simplified by assuming homogeneous bone support and by reducing the complexity of the trabecular structure, which may influence stress transmission within the bone. In clinical settings, teeth can transmit stresses through the alveolar bone, which is made up of both cortical and trabecular components, and this may lead to variations in the stress distribution patterns compared with those observed in the model. Therefore, the findings should be interpreted with caution from a clinical perspective, and future studies should be designed to incorporate heterogeneous tissue structures and dynamic loading conditions. In this study, only 300 N static vertical loading was applied. However, in clinical conditions, dynamic and lateral forces also play a significant role during mastication. Future studies incorporating these forces and tissue anisotropy into more complex FEA models will enhance the clinical relevance and validity of the findings. Additionally, in this study, the model was generated using only one extracted maxillary first molar, which represents a limitation as it does not account for biological variability between samples. Since the FEA method is a numerical simulation, the stress values were not statistically analyzed; however, the obtained results were found to be consistent with previous studies.

This FEA study demonstrated that palatal root amputation results in the greatest increase in stress and emphasizes the critical role of the palatal root in maintaining tooth stability. For clinical success, preservation of the tooth structure and supporting tissues and careful management of occlusal forces are essential. These findings highlight the contribution of preserving the natural tooth structure to the clinical decision-making process, while addressing the biomechanical disadvantages of root amputation. The numerical FEA findings obtained from this study represent theoretical stress distribution and should not be directly correlated with clinical outcomes.

## 5. Conclusions

Within the limitations of this study, we make the following conclusions:

- Root resection weakens the tooth’s structural integrity and significantly affects stress distributions.
- The highest stress in the dentin tissue and root canal filling material was observed following palatal root resection, followed by mesial and distal root resections, respectively.
- Stress distribution patterns resulting from mesial and distal root resections were similar, and the stress values were relatively close.

These findings highlight the biomechanical importance of root selection when evaluating the feasibility of resection procedures. Clinically, preserving this root whenever possible or using biomimetic restoration techniques that mimic natural load transfer can increase the long-term stability of the tooth. In this study, palatal root resection was found to produce the highest tensile stresses in dentin and alveolar bone, suggesting a potential biomechanical disadvantage. In addition, these findings underscore the clinical importance of preserving the palatal root whenever feasible and highlight the necessity of restorative planning aimed at achieving balanced occlusal load distribution. Both biological and biomechanical factors should be considered when making decisions regarding root amputation. Since the FEA method provides a theoretical model, further in vitro and in vivo studies incorporating dynamic forces are recommended to validate the results.

## Figures and Tables

**Table 1:** Number of nodes and elements in groups.

	Model No.	Nodes	Elements
Endodontically treated tooth (not resected)	Model 1	861,872	595,449
Palatal root resection	Model 2	732,494	504,358
Disto-buccal root resection	Model 3	774,613	533,668
Mesio-buccal root resection	Model 4	704,221	484,328

**Table 2:** Elastic properties of the materials used in the stress analysis models.

Materials	Elastic Modulus (E) (MPa)	Poisson’s Ratio (X)
Enamel [23]	84,100	0.33
Dentin [24]	18,600	0.31
Composite resin [25]	16,400	0.28
Gutta-percha [26]	140	0.45
Periodontal ligament [26]	0.0689	0.45
Cortical bone [27]	13,700	0.30
Spongy bone [27]	1370	0.30
MTA [28]	11,760	0.314
Flowable Resin [29]	7500	0.25

**Table 3:** Maximum tensile stress values (MPa) observed in dentin tissue and root canal filling material in different models.

	Enamel	Dentin	Root Filling/Plug Material	Alveolar Bony
Not resected	2.13	8.14	1.86 (Gutta Percha)	0.83
Palatal root resection	5.12	15.86	18.33 (MTA)	14.42
DB root resection	3.13	11.63	13.10 (MTA)	11.74
MB root resection	3.72	12.22	14.09 (MTA)	12.34

## Data Availability

No new data were created.
